# A Systematic Literature Review on Pre-exposure Prophylaxis as a Strategy for HIV Risk Reduction in the Middle East

**DOI:** 10.7759/cureus.80842

**Published:** 2025-03-19

**Authors:** Mas Chaponda, Ahmed A AlHammadi, Ali Alsaeed, Batool Ali, Jameela Al Salman, Roaa S Alosaimi

**Affiliations:** 1 Infectious Department, Communicable Diseases Center, Qatar University, Doha, QAT; 2 Department of Internal Medicine, AlRahba Hospital, Abu Dhabi Health Services, Abu Dhabi, ARE; 3 Infectious Diseases, Emirates Infectious Disease Society, Emirates Medical Association, Dubai, ARE; 4 Infectious Disease Division, Department of Internal Medicine, Dammam Medical Complex, Eastern Health Cluster, Dammam, SAU; 5 Infectious Diseases, HIV Center of Excellence, East Jeddah Hospital, Jeddah, SAU; 6 Infectious Diseases, Arabian Gulf University, A'Ali, BHR; 7 Geriatric Medicine, King Hamad American Mission Hospital, A’Ali, BHR

**Keywords:** hiv prevention, human immunodeficiency virus (hiv), middle east and north africa (mena), pre-exposure prophylaxis (prep), prophylaxis

## Abstract

Pre-exposure prophylaxis (PrEP) has emerged as a crucial tool in HIV prevention globally, yet its implementation in the Middle East and North Africa (MENA) region faces unique challenges. PrEP is expected to be effective in reducing HIV transmission. However, there is limited comprehensive data about its awareness, utilization, and effectiveness within MENA countries. The region's distinct cultural, social, and healthcare system characteristics create specific barriers that must be addressed. This study aims to evaluate PrEP's value and effectiveness and to develop targeted recommendations for overcoming barriers and expanding PrEP programs to better serve the region's specific needs and populations at risk. We searched PubMed, Scopus, and Web of Science databases through October 2024, using keywords related to HIV, PrEP, and the MENA region. Eligible studies included peer-reviewed clinical research on PrEP use in MENA countries, focusing on high-risk populations. Two independent reviewers screened titles, abstracts, and full texts using the Rayyan software, with disagreements resolved by a senior reviewer. Studies across the MENA region showed varying levels of PrEP awareness and willingness to use, with MSM communities showing higher interest. While PrEP proved effective for HIV prevention when properly used, implementation faced barriers, including costs, stigma, and accessibility. Despite the potential of PrEP implementation in HIV prevention, its efficacy in the MENA region remains unproven due to a lack of clinical studies. Successful implementation of PrEP in this region requires addressing key challenges, including financial accessibility, awareness, stigma, and healthcare integration. Morocco's pilot program serves as a promising example, but broader adoption must focus on improving accessibility and affordability. Future efforts should tailor interventions to meet the needs of at-risk populations, with an emphasis on enhancing adherence and retention rates to ensure effectiveness in diverse MENA settings.

## Introduction and background

Human immunodeficiency virus (HIV) is a global health problem, causing significant morbidity and mortality. It weakens the immune system, leaving the human body vulnerable to infections and some cancers [1]. The global newly infected cases in 2023 was 1.3 million, representing a 39% decrease in new cases since 2010 [2,3]. However, this decline falls short of the target to bring new cases below 370,000 by 2025 [2]. However, in the Middle East and North Africa (MENA) region, the incidence of HIV has increased and reached 23,000 new cases and 6,200 related deaths in 2023 [2]. Thus, there is an unmet need to improve HIV prevention strategies.

Oral pre-exposure prophylaxis (PrEP) represents a promising prevention tool with proven high efficacy in clinical trials across different populations, especially when adherence is consistently high [4]. In 2015, the World Health Organization (WHO) recommended oral PrEP as an additive preventive option in high-risk populations [5]. This recommendation was updated in 2019 to include on-demand or event-driven PrEP and in 2021 to include vaginal ring PrEP [6,7]. Based on the previous recommendations, by 2018, a total of 19 high-income countries and 21 low- or middle-income countries had either implemented or were planning to implement PrEP programs [8].

Despite this guidance and varied prevention strategies, PrEP utilization remains low [9,10]. This may be due to multiple barriers, including perceived side effects, cost, anticipated stigma, and poor awareness [11-14]. A recent meta-analysis across various populations and cultural contexts, including those in the MENA region, reported that awareness of PrEP reached 50% among men who have sex with men (MSM), with 58.6% expressing willingness to use it. Awareness has increased over time; however, stigma remains a significant barrier to HIV prevention among MSM in the MENA region [15].

There is a lack of comprehensive data on PrEP awareness, utilization rates, and culturally relevant barriers across MENA countries. Therefore, we aim to provide a systematic literature review to summarize the evidence about the value and effectiveness of PrEP as an HIV prevention tool within the MENA region. We also aim to provide some recommendations for overcoming these barriers and scaling up PrEP programs to meet the unique needs of this region.

## Review

Methods

We followed the Cochrane Handbook for Systematic Reviews in conducting our study [16]. The results are reported following the Preferred Reporting Items for Systematic Reviews and Meta-Analyses (PRISMA) guidelines [17].

Data Sources and Search Terms

We searched different databases, including PubMed, Scopus, and Web of Science, until October 2024. The search strategy incorporated keywords related to "HIV", "PrEP", and "MENA". Detailed search strategies for each database are provided in the Appendix.

Eligibility Criteria and Study Selection

We included any peer-reviewed primary clinical study related to the use of PrEP in the MENA region, incorporating populations at risk for HIV infection (including high-risk groups such as MSM, sex workers, people who inject drugs, and serodiscordant couples). In addition, studies examining the knowledge and awareness of PrEP use among healthcare workers or the general population were included. Animal studies, conference abstracts, and non-English studies were excluded from consideration. The identified citations from the above-mentioned databases were exported for further evaluation. Two independent reviewers performed the title, abstract, and full-text screening using the Rayyan software (Rayyan Systems Inc., Cambridge, MA) [18]. In case of any disagreements between the reviewers, a senior reviewer was consulted to resolve the issues.

Data Extraction

For each eligible study, we extracted the following data where available: study ID, country, sample size, study design, study duration, inclusion and exclusion criteria, age, gender distribution (male percentage), key population (e.g., men who have sex with men (MSM), female sex workers (FSW), baseline HIV risk factors, PrEP usage details, PrEP intervention type and dosage, assessed outcomes, study conclusions, adherence rates, acceptability and feasibility, awareness levels, willingness to use PrEP, identified barriers, and funding sources. Two independent reviewers did data extraction to ensure accuracy.

Data Synthesis

Due to the lack of consistent study designs, outcomes, and population characteristics across the included studies, we narratively reported the evidence. Key outcomes, including effectiveness, awareness, willingness, and cost-effectiveness, were summarized from the different included studies to provide a comprehensive overview of PrEP use in the MENA region. In addition, we assessed each included study individually, highlighting the main findings and key points reported in each.

Results

Study Selection

**Figure 1:**
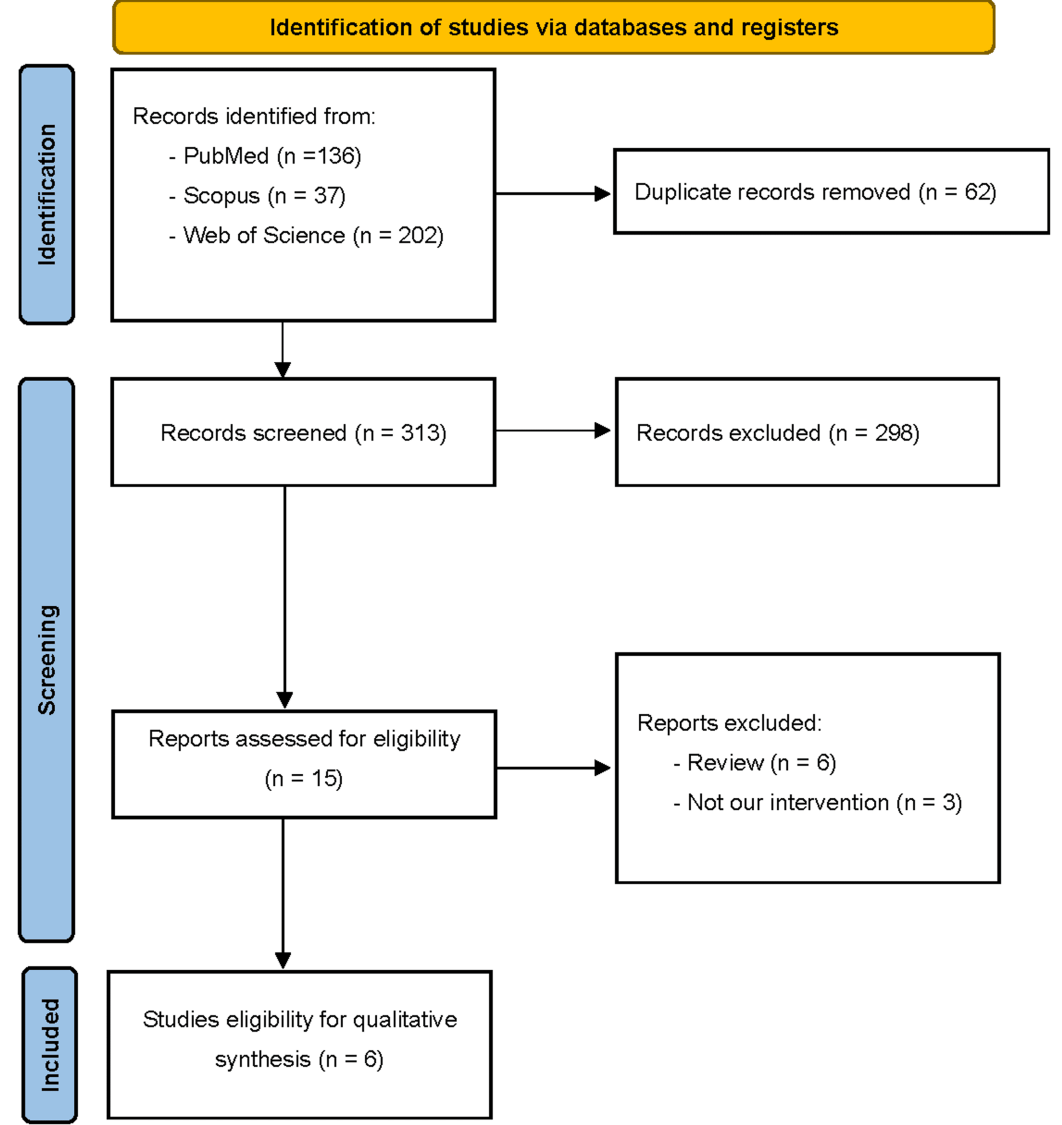
Preferred Reporting Items for Systematic Reviews and Meta-Analysis (PRISMA) flow diagram

After discarding any duplications, 313 articles were screened for title and abstract screening, resulting in the inclusion of six [19-24] articles after the performance of full-text screening (Figure 1). Across four cross-sectional studies, in addition to two observational cohort studies, no RCTs were included due to the lack of data in the MENA region.

Study Characteristics

A total of 6,021 participants were included from six different studies; the studies varied in duration as the lowest-duration study was one month while the highest-duration study was 10 months. Two of the included studies were in Lebanon, two studies in Morocco, and two in Turkey. Table 1 shows a summary of the included studies. The most commonly targeted population was MSM. The baseline characteristics of the enrolled patients are provided in Table 2. The majority of the included studies assessed the awareness of PrEP, sexual attitude, and willingness to use PrEP. While some studies showed that most subjects adhered to PrEP, other studies reported low adherence rates [19-24].

**Table 1:** Summary of the included studies. Abbreviations: FSWs: female sex workers; MSM: men who have sex with men; PrEP: pre-exposure prophylaxis; HBV: hepatitis B virus; STI: sexually transmitted infection; PEP: post-exposure prophylaxis; KLIMIK: Turkish Society of Infectious Diseases and Clinical Microbiology

Study ID	Country	Sample size	Study design	Study duration	Inclusion criteria	Funding source
Azzi (2024) [19]	Lebanon	410	Cross-sectional study	5 months	Lebanese population who have access to WhatsApp and the internet	None
Moussa (2024) [22]	Morocco	-FSWs: 10	Cross-sectional study (using semi-structured interviews)	2.5 months	(a) Being assigned female sex at birth	Agence Française d’Expertise Technique Internationale
					(b) 18 years of age or older	
					(c) Having had sex with men in the last 6 months in exchange for money or material goods	
		- key stakeholders: 28			(d) Having resided in Morocco for the last 6 months	
					(e) Being HIV-negative	
					(f) Consenting to participate in the study.	
Moussa (2024) (2) [21]	Morocco	396 people:	Prospective cohort study	Approximately 10 months		the Global Fund to Fight AIDS, Tuberculosis and Malaria support program to fight HIV/AIDS.
					(a) Being HIV negative	
					(b) High risk of sexual acquisition of HIV infection, one of the criteria below is sufficient:	
		(97 FSW and 299 MSM)			- Condomless sex with at least 2 different partners over the past 6 months	
					- Having at least one STI episode in the past 12 months	
					- Having multiple courses of postexposure prophylaxis (PEP) within the prior 12 months	
					- Use of alcohol and substances during intercourses	
					- Likely to join the study agrees to come monthly for the follow-up.	
Nazli (2022) [23]	Turkey	4761	Cross-sectional study	Approximately 2 months	(a) Age: 18 years or older	None
					(b) HIV Status: HIV-negative	
					(c) HBV Status: HBV-negative	
					(d) Willingness to participate	
					(e) Regular attendance	
Cimen (2020) [20]	Turkey	190	Cross sectional study	1 Month	(a) Respondents must be members of the Turkish Society of Infectious Diseases and Clinical Microbiology (KLIMIK).	None
					(b) The survey aimed to gather responses from active members of KLIMIK, which had nearly 2100 active members at the time of data collection.	
					(c) Participants had to provide completed surveys that included the primary interests of the study, specifically attitudes about PrEP and PrEP recommendation.	
Storholm (2021) [24]	Lebanon	226	Observational cohort study	8 months	(a) Being assigned male sex at birth and currently male-identified	The US National Institute of Mental Health with addition funding for Dr. Storholm provided by the US
					(b) Age 18 to 29 years	
					(c) Fluent in English or Arabic	
					(d) Residing in greater Beirut	
					(e) Having had oral or anal sex with a man in the past 12 months.	

**Table 2:** Baseline characteristics of the enrolled patients in the included studies. Abbreviations: FSWs: female sex workers; MSM: men who have sex with men; PrEP: pre-exposure prophylaxis; STD: sexually transmitted disease; STI: sexually transmitted infection; NA: not available/not applicable

Study ID	Age (years), mean (SD)	Gender (Male n %)	Key population (e.g., MSM, FSW)	Baseline HIV risk factors	PrEP usage
Azzi (2024) [19]	27.39	203 (49.5%)	General population	(a) Participants with multiple partners: 12 (2.9%)	5 (5.5%)
				(b) Participants with a history of STD diagnosis: 3 (1.5%)	
				(c) Participants reporting drug use: 54 (13.2%)	
				(d) Regular alcohol consumption: 45 (11%)	
				(e) Daily Tobacco/Hookah usage: 56 (13.7%)	
				(f) Daily E-Cigarette usage: 35 (8.5%)	
				(g) Practice of safe sex (not "all the time") total: 130 (67% of sexually active participants)	
Moussa (2024) [22]	37.20 (9.53)	0 (0%)	-FSWs: 10	NA	NA
			- Key stakeholders: 28, including 10 physicians who prescribed PrEP, eight policymakers, and 10 community advocates.		
Moussa (2024) (2) [21]	MSM: 30.4 years	299 (75.5%)	97 FSW	NA	320 (86%)
	FSW: 38.2 years		299 MSM		
Nazli (2022) [23]	30.52 (9.5)	4761 (100%)	MSM	(a) STI diagnosis in the last 3 months 297 (8.6%)	83 (1.7%)
				(b) Chemsex in the last 3 months 224 (6.6%)	
Cimen (2020) [20]	24–30 y: 43 (22.6%)	63 (33.2)	Infectious diseases physicians	NA	NA
	31–40 y: 95 (50%)				
	41–50 y: 38 (20%)				
	>50 y: 14 (7.4%)				
Storholm (2021) [24]	<25 y: 134 (61.5%)	226 (100%)	Young MSM	NA	NA
	>25 y: 84 (38.5%)				

The levels of awareness among patients with high-risk factors for HIV were inconsistent, and the majority of global studies indicate that awareness remained low in this population. There is a critical need to enhance awareness of PrEP methods among these individuals. The main findings of the included studies are shown in Table 3.

**Table 3:** Main findings of the included studies. Abbreviations: PrEP: pre-exposure prophylaxis; NA: not available; MSM: men who have sex with men; FSWs: female sex workers; STI: sexually transmitted infection; HIV: human immunodeficiency virus; HBV: hepatitis B virus; LAI: long-acting injectable; YMSM: young men who have sex with men

Study ID	Outcomes assessed	Conclusion	Adherence rates	Acceptability and feasibility	Awareness	Willingness to use	Barriers
Azzi (2024) [19]	PrEP awareness, willingness, barriers	Highlights the need for tailored education and community initiatives in Lebanon	NA	NA	91 (22.2%)	52 (57.1%)	1. Low awareness: Only 22.2% are aware of PrEP.
							2. Misconceptions: 34% mistakenly believe PrEP protects against all STIs.
							3. Dosing confusion: Only 22% understand daily vs. on-demand dosing.
							4. Perceived low risk: 64% of non-users see low personal risk for HIV.
							5. Stigma: 16% fear stigma and discrimination related to PrEP use.
							6. Side effect concerns: 16% worry about potential side effects.
							7. Limited provider info: Few receive guidance (16% from specialists, 2% from GPs).
							8. Financial barriers: Greater willingness if PrEP is free and pharmacy-accessible.
							9. Low preventive behavior: Only 16.5% had HIV testing in the past year.
Moussa (2024) [22]	PrEP awareness, challenges, delivery preferences, perceived benefits, and risks of PrEP	Emphasizes acceptability issues and preference for LAI PrEP among FSWs in Morocco	NA	Acceptability:	Low Awareness	Mixed Willingness	1. Stigma and social perception
				Stigma, cultural beliefs, and knowledge gaps reduce PrEP acceptability; high interest in long-acting injectable (LAI) PrEP for discretion.			2. Economic barriers
				Feasibility:			3. Daily pill adherence
				Economic barriers and adherence issues limit feasibility; LAI PrEP and peer education can improve access and support.			4. Lack of awareness and knowledge
							5. Cultural attitudes toward medication
							6. Inconsistent condom use
							7. Healthcare provider knowledge gaps
							8. Preference for alternative PrEP forms
							9. Legal and social vulnerability
							10. Transportation and access issues
Moussa (2024) (2) [21]	PrEP uptake, retention, safety	The community-based PrEP program shows high interest among MSM and FSW in Morocco.	moderate retention rate of 37%	High acceptability and feasibility are based on high uptake.	A high level of interest and awareness is based on high uptake.	NA	NA
Nazli (2022) [23]	PrEP knowledge, use, willingness, STI testing, Chemsex, sexual happiness levels	moderate awareness and willingness for PrEP among MSM in Turkey	NA	NA	1904 (40.5%)	1468 (39.4%)	1. Lack of awareness
							2. Negative perceptions and stigma
							3. Limited access to healthcare
Cimen (2020) [20]	1. Knowledge and sources of PrEP	Recommends guidelines to support healthcare professionals’ attitudes on PrEP	NA	NA	NA	NA	NA
	2. Attitudes toward PrEP						
	Note: The population is healthcare professionals						
Storholm (2021) [24]	1. Knowledge of HIV risk	Greater peer judgment about sexual risk was linked to higher willingness to take PrEP among YMSM, possibly because those engaging in riskier behaviors are more aware of their own risk due to peer feedback.	NA	The majority presented high acceptability.	28.10%	55.50%	1. Stigma
							2. Insurance and financial constraints
	2. Awareness of PrEP						3. Access to healthcare
							4. Prescription coverage

Systematic Review of the Literature

Effectiveness of PrEP: In an online survey in Turkey, the results showed that 85.3% of the participants found that PrEP was effective as a preventive measure [20]. Otherwise, no data were reported regarding the effectiveness in the other included studies. However, the key point reported for increasing the effectiveness of PrEP is strict adherence, as taking the drug daily as prescribed would almost result in complete protection against HIV. In addition, missing doses can reduce its effectiveness significantly. The protection is limited to HIV, and it does not protect against other sexually transmitted infections (STIs), so it is often recommended alongside condom use.

Awareness of PrEP: In a study from Lebanon, the awareness level of PrEP was moderate within the Lebanese community, although there were some concerns about this study's generalizability [19]. Only 22.2% of individuals reported being aware of PrEP, indicating varied levels of understanding regarding its purpose and usage. Among those familiar with PrEP, 34% mistakenly believed it protected against all STIs. In addition, 45% knew that PrEP was available in Lebanon [19]. Increasing awareness could be crucial in expanding PrEP’s impact as a preventive measure [19]. The awareness among FSWs was lacking in the Moussa 2024 et al. [22] study that showed the importance of PrEP promotion and uptake as a significant way to prevent HIV. An awareness campaign is needed to widely promote PrEP, remove stigmatization and the misconception of these drugs as experimental, with extensive advertising about the medication, including its safety and benefits. Pharmacies could also play a key role in raising awareness by highlighting PrEP’s availability and advantages [22]. In another study from Morocco, the awareness level was high in level and based on high uptake [21].

In Turkey, testing PrEP awareness among MSM found that the degree of awareness was moderate (40.5%) [23]. When specifying the population, Arabic-speaking respondents were associated with a lower level of awareness (27.6%) [23]. When assessing the awareness of the MSM population in Beirut, 57.3% of the population didn’t even know what PrEP was [25].

Willingness to use PrEP: In the Lebanese community, out of those aware of PrEP, Only 57.1% were willing to use PrEP, while 5.5% had tried it [19]. Another study from Lebanon in MSM showed that the willingness to take PrEP was more than half of the included population, with a percentage of 55.5 [24]. The study by Moussa 2024 et al. [22] showed a varied response in willingness, as many FSWs are willing to use PrEP despite the different concerns such as social stigma, financial difficulties due to the cost of transportation in spite the free program, and challenges with taking a daily pill and staying adherent. The study explained this variance on the individualized personal situation such as the temporary work status and the belief of condom effectiveness alone. In Turkey, the willingness to use PrEP in the capital in Istanbul was 39.6%, which was similar to other cities as the willingness to take PrEP was 39.4% [23].

Cost-effectiveness of PrEP: In Turkey, they reported concern about cost-effectiveness as the service would have a high impact on the budget of the associated population [20]. Moussa et al. (2024) [22] found that despite PrEP being provided at no cost through the Association de Lutte Contre le Sida (ALCS)'s PrEP clinics in Morocco, transportation costs created a significant financial barrier for many potential users, particularly FSWs. The researchers observed that most FSW participants lived in poverty and struggled to afford travel expenses to reach PrEP clinics, sometimes even preventing them from accessing free condoms [22].

Evaluation of the included studies: A Lebanese study [19] assessed PrEP awareness and willingness to use among the general population. The study found that only 22.2% of participants were aware of PrEP, and 57.1% expressed willingness to use it, but only 5.5% actually used it. The main key data sources were the Internet and social media. Healthcare professionals had a minimal role. This study shed light on the barriers, such as perceived low risk (64%), and concerns about discrimination or side effects. They also correlated awareness and willingness to demographic, behavioral, and attitude factors. While awareness was negatively associated with a younger population, reflecting less awareness and positively in individuals with multiple sexual partners, it was not statistically significant with MSM. Afterward, excluding confounding variables by logistic regression revealed a sexual orientation significant association. In addition, willingness was more reported with younger individuals and upon discussing with friends or family or having access to enough information. Finally, the study suggests that improving education alongside increasing the pharmaceutical accessibility of PrEP by reducing financial barriers would enhance the uptake within Lebanese society.

A Turkish study [20] of 209 infectious diseases physicians regarding their attitudes and knowledge of PrEP is different from other included studies as this study focused on medical professionals. Most participants were females (66.3%). About 85.3% believed PrEP was efficient, 66.3% considered it safe, and 24.2% had recommended it in their clinical practice. The main concerns about PrEP included potential increases in sexually transmitted infections (88.1%), cost-effectiveness pressures (70.1%), and doubts about its effectiveness in HIV transmission (55.2%). The physicians rated their own knowledge regarding PrEP as low and suggested the development of national guidelines to help address their concerns and change attitudes toward PrEP implementation in Turkey.

**Figure 2:**
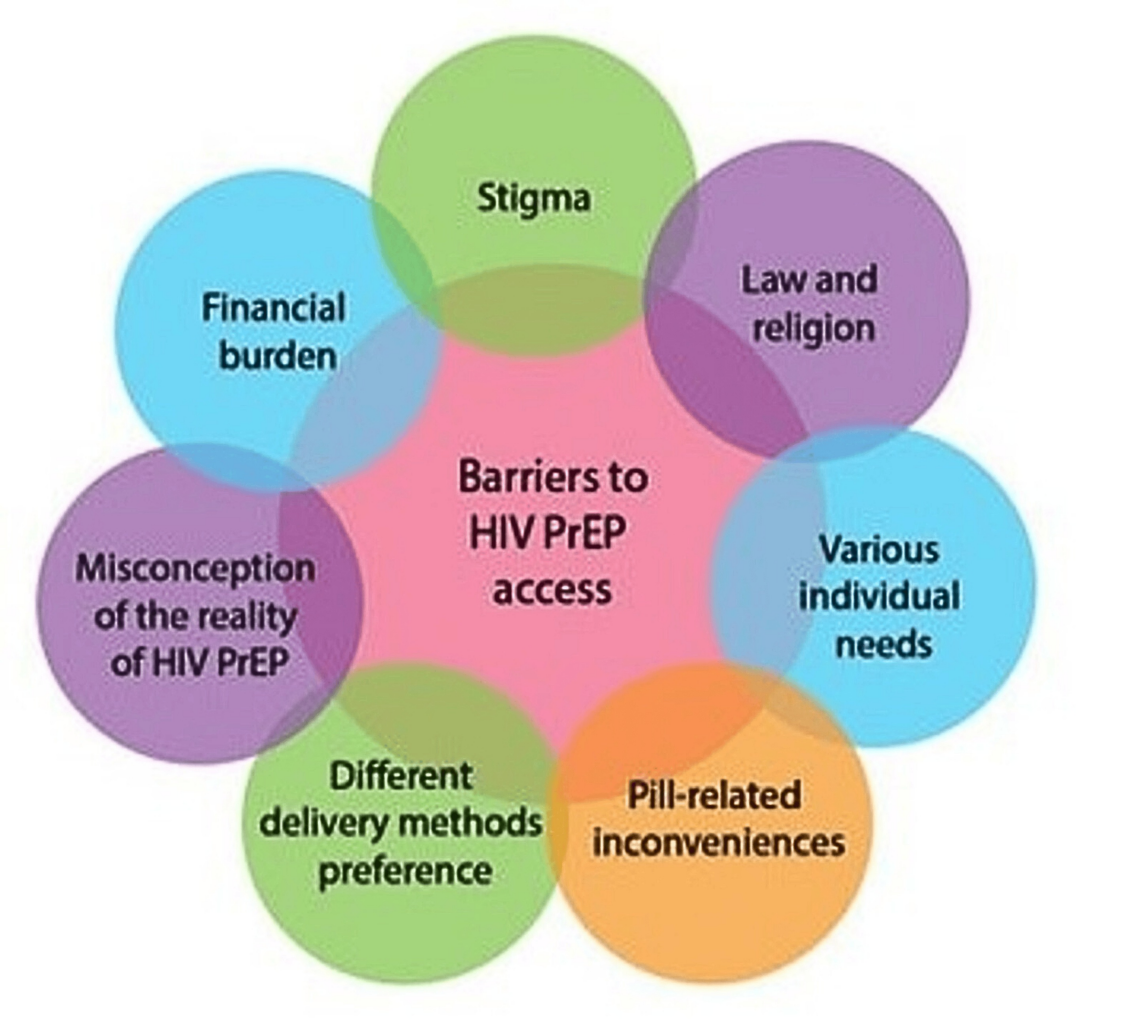
Barriers to HIV PrEP in the MENA region. HIV PrEP: human immunodeficiency virus pre-exposure prophylaxis, MENA: Middle East and North Africa

A Moroccan study [22] examined the barriers to HIV PrEP access by interviewing FSWs, physicians, policymakers, and community advocates. The study identified several barriers: 1) PrEP stigma: FSWs were afraid to be stigmatized as HIV patients if discovered using the pills, which could result in violence from their clients physical or sexual. 2) Law and religion: Commercial sex work is prohibited and illegal, which hinders accessibility of these groups to HIV/PrEP program. 3) The wide variation of individual needs: While some FSWs were convinced in the efficiency of condoms alone, others thought the combination of pills and condoms were better. By contrast, some females refused the drugs based on their personal temporary sex work, and others feared pregnancy over HIV, underscoring the need for tailored prevention for each subject at risk. 4) The misconception of the reality of HIV PrEP: It appeared that many FSWs believed that these drugs are only experimental, based on their absence in pharmacies and the low level of awareness among the society, including healthcare workers. 5) Financial burden: Although PrEP was free of charge in the Morrocco program, FSWs faced the problem of transportation costs. 6) Pill-related inconveniences: The need to take the pills every day and their side effects, such as stomach upset, made it difficult for these women to adhere to the pills in the long term. 7) The preferences for different PrEP delivery methods: FSWs reported preference of long acting injectables (LAI) over the pills or vaginal rings due to adherence and sex work interference issues (Figure 2). This study's main finding suggests that HIV prevention approaches should be more suitable for users' lifestyles and circumstances. This study is the first study to assess the PrEP barriers among FSWs in the MENA region, which shows the importance of this study’s findings and the need to perform similar larger studies.

A second study from Morocco [21] assessed PrEP in MSM and FSW across three cities with high HIV prevalence. The study achieved a high PrEP uptake rate of 86% (86.6% for MSM and 83.5% for FSW). The overall retention rate was 37%, and the retention rate after the critical three-month period was significantly higher at 78% (81% for MSM and 62% for FSW). The MSM showed better retention rates than the FSW. The study demonstrated PrEP's feasibility and acceptability in Morocco's cultural context, making it a potential model for other Middle Eastern and North African countries.

A study in Turkey [23] investigated the awareness of PrEP among MSM by an anonymous self-completed questionnaire. The questionnaire comprised 20 questions on HIV and STI awareness and willingness to use PrEP. The study found that 40.5% of respondents knew about PrEP, with most learning about it online (44.5%), while only 2.5% had used PrEP in the past year and 1.7% were current users. Despite PrEP not being licensed in Turkey at the time of the study, 39.4% expressed willingness to use it. Arabic speakers showed lower awareness of PrEP and HIV testing rates but a higher willingness to use PrEP in the future (61.1% vs. 38.2%). In addition, younger individuals also expressed less awareness and previous HIV testing in the last six months, but less willingness to take the drugs in the future. This study concluded a high degree of willingness among MSM in Turkey, particularly with the Arabic population, suggestingthat solving the availability problem could decrease the incidence of HIV.

A Lebanese study [24] examined the willingness of young MSM to take PrEP in Beirut. This is the first study in the MENA region to evaluate this outcome. Over half of the participants (55.5%) reported a willingness to take PrEP, with greater willingness associated with HIV risk knowledge, PrEP awareness, and relationship status. After performing logistic regression analysis, the study found that substance use prior to or during sexual activity has a significant correlation with PrEP willingness, whereas PrEP knowledge had a marginal correlation. In addition, the study found that PrEP remained largely inaccessible and unaffordable in Beirut and the broader region. The study recommended that the ministry in Lebanon improve PrEP accessibility and affordability.

Discussion

Studies across multiple Middle Eastern countries showed varying levels of PrEP awareness and implementation. Awareness levels ranged from low (22.2% in Lebanon's general population) to moderate in Turkey (40.5% among MSM). Willingness to use PrEP varied between 39.4% and 57.1% across different populations, with higher rates among MSM communities. The effectiveness of PrEP was not an outcome of interest in most of the studies. However, when reported, it showed high protection rates against HIV when properly adhered to. Nevertheless, significant barriers were identified, including high costs, social stigma, and accessibility issues.

Morocco showed promising results with high uptake rates (86%) among both MSM and FSW populations, especially when fully supported by funds from the government. It now has an HIV program that could represent an example for the other MENA region countries.

Studies from Turkey reported moderate awareness (40.5%) and willingness (39.4%) to use PrEP in the future among MSM, along with high willingness (61.1%) in the Arab respondents despite their low awareness. In addition, they reported a consistent willingness across cities despite the absence of a license, suggesting great uptake potential if accessibility was addressed. On the other hand, Lebanon’s general population was less aware (28.1%), but their MSM group was more willing (55.5%) when supported by peer discussion. This can be mainly attributed to the misconception about PrEP use in addition to factors such as stigma and cost.

When comparing the awareness in the MENA region with other countries, we can see that Ferrand et al. (2023), who conducted their study in the US, found that the awareness level was 29% of their included population [25]. While Wang et al. 2022 found that awareness in the Netherlands ranged from 78%-91% [26]. When categorizing countries according to economic levels, Yi et al. (2017) found that awareness in low- and middle-income countries was lower than in high-income countries [27].

Previous studies investigated the effectiveness of PrEP and found a significant reduction in HIV infection that reached 51% (4). In studies with moderate adherence levels, PrEP significantly reduced the risk of infection; however, it demonstrated no effect in studies characterized by low adherence [4]. In addition, our research aligns with the previous literature in terms of effectiveness, cost, and cost effectiveness. A comprehensive literature systematic review by Bozzani et al. [28]. evaluated the cost and cost effectiveness of the biomedical interventions on HIV, including vaccines and medications. They ended up having most of the studies conducted in low and middle-income countries (LMICs) and investigating PrEP regimens, matching our population and intervention. A conclusion of the valid and effective application of PrEP over the high-risk population was drawn. In addition, the cost beyond the medication, such as expenses related to program administration, training, adherence support, monitoring, and integration with other healthcare service,s alongside the limited data from various settings and the cost of outreach to the targeted population have also limited the conclusion of feasible practical implementation and a scale model distribution. LAI was also a preference in a US Preventive Services Task Force systematic review by Chou et al., alongside the risk reduction of HIV by 54% compared to placebo or no PrEP [29]. This aligns with our result of LAI preference by FSWs in the Morocco community. 

Giddings et al. [1] pointed out the absence of models that can evaluate the synergistic effects of HIV prevention interventions combinations such as PrEP and condoms in real scenarios because most of the studies conducted assessed each intervention solely. This interaction neglection could amplify the overall effectiveness and deprive us of he chance of outcome enhancement in regions like MENA where stigma, accessibility, and awareness issues are present.

The National HIV Management Guideline by the United Arab Emirates Ministry of Health and Prevention acknowledged the effectiveness of PrEP on adherence, especially when condoms are not consistently used, supporting the call for tailored interventions [30]. It also advised to educate users that PrEP is not meant to prevent all the STIs, supporting the need for complementary interventions like condom use and STI testing. This is in agreement with the Saudi Arabia HIV guideline, which also emphasizes the use of PrEP in high-risk populations, the importance of adherence, and the necessity of integrating PrEP with broader sexual health strategies [31]. Both guidelines collectively call for holistic approaches to HIV prevention, combining biomedical interventions like PrEP with behavioral and systemic support to address gaps in awareness, adherence, and STI prevention in the MENA region.

In addition, the Saudi Arabia Guideline for HIV treatment further outlined that when discontinuing PrEP, patients should continue for two days after the last HIV exposure or seven days for daily users (30). It stated that healthcare providers must document HIV status, reason for discontinuation, medication adherence, and sexual risk behavior. Follow-up HIV testing using a fourth-generation test should be conducted within eight weeks of discontinuation (30). These guidelines and recommendations support our call for national policy guidelines integration where evidence-based care is needed to maximize the benefits.

Regarding accessibility, non-traditional PrEP delivery methods were investigated by a US systematic review, which found a probability of access increase if we overstep the health care system [32]. For example, easing pharmacists’ prescriptions and mail-in laboratory testing can overcome society related factors such as stigma and accessibility.

Our study has multiple strengths, as we are the first study to evaluate PrEP against HIV patients in the MENA region. We included all the available data despite the scarcity of data. We covered different aspects of the topic to ensure the best understanding; we covered the topic from the aspect of MSM, FSW, and medical professionals.

We have several limitations, as we relied on observational study designs as there are no RCTs in the MENA region. We included only three MENA region countries, which may limit the generalizability of our findings and may not reflect the diversity of the region.

We recommend that future researchers set RCTs to investigate the effectiveness of the PrEP. We also recommend reducing the total cost of the PrEP by finding cheaper alternatives and integrating these regimens into the national health insurance package, which would help alleviate these financial burdens and improve accessibility for potential users. In addition, we suggest using targeted campaigns for high-risk populations such as MSM and FSW and going further with non-traditional methods that can solve the accessibility problems like pharmacy-based access and telemedicine.

## Conclusions

Despite the potential of PrEP in HIV prevention, its efficacy in the MENA region remains unproven due to a lack of clinical studies. PrEP implementation faces multiple challenges that need to be addressed systematically. The key areas requiring attention are reducing financial barriers through cost reduction or insurance coverage, increasing awareness through targeted educational campaigns, addressing social stigma, and improving healthcare system integration, such as integrating PrEP into the established STI or HIV clinics. The established success found in the HIV program, despite cultural challenges, provides a potential model for MENA countries. Future interventions should focus on making PrEP more accessible and affordable while considering the specific needs of different at-risk populations, particularly MSM and FSW communities, and developing strategies to improve long-term adherence and retention rates.

## Appendices

**Table 4:** Search results Total results: 375, duplicates: 62, total after duplicate removal: 313, full-text screening: 15, final included: 6

Database	Search strategy	Number of results
PubMed	("HIV" OR "Human Immunodeficiency Virus") AND ("pre-exposure prophylaxis" OR "PrEP") AND ("Algeria" OR "Bahrain" OR "Egypt" OR "Iran" OR "Iraq" OR "Jordan" OR "Kuwait" OR "Lebanon" OR "Libya" OR "Morocco" OR "Oman" OR "Qatar" OR "Saudi Arabia" OR "Syria" OR "Tunisia" OR "United Arab Emirates" OR "Palestine" OR "Yemen" OR "Sudan" OR "Turkey" OR "Middle East" OR "North Africa" OR "MENA" OR "Gulf Region" OR "Arab region")	136
SCOPUS	(TITLE-ABS-KEY ("HIV" OR "Human Immunodeficiency Virus")) AND (TITLE-ABS-KEY ("pre-exposure prophylaxis" OR "PrEP")) AND (TITLE-ABS-KEY ("Algeria" OR "Bahrain" OR "Egypt" OR "Iran" OR "Iraq" OR "Jordan" OR "Kuwait" OR "Lebanon" OR "Libya" OR "Morocco" OR "Oman" OR "Qatar" OR "Saudi Arabia" OR "Syria" OR "Tunisia" OR "United Arab Emirates" OR "Palestine" OR "Yemen" OR "Sudan" OR "Turkey" OR "Middle East" OR "North Africa" OR "MENA" OR "Gulf Region" OR "Arab region"))	37
WOS	ALL=("HIV" OR "Human Immunodeficiency Virus") AND ALL=("pre-exposure prophylaxis" OR "PrEP") AND ALL=("Algeria" OR "Bahrain" OR "Egypt" OR "Iran" OR "Iraq" OR "Jordan" OR "Kuwait" OR "Lebanon" OR "Libya" OR "Morocco" OR "Oman" OR "Qatar" OR "Saudi Arabia" OR "Syria" OR "Tunisia" OR "United Arab Emirates" OR "Palestine" OR "Yemen" OR "Sudan" OR "Turkey" OR "Middle East" OR "North Africa" OR "MENA" OR "Gulf Region" OR "Arab region")	202
